# TRALI in Perioperative Period-A Case Report

**Published:** 2009-04

**Authors:** G Ilango, S Senthilkumar, N Sambanthan

**Affiliations:** 1 Assistant Professor, Department of Anaesthesiology, SRM Medical College Hospital and Research Centre, Potheri, Chennai- 603203, Tamilnadu, India; 2 Senior Resident, Department of Anaesthesiology, SRM Medical College Hospital and Research Centre, Potheri, Chennai- 603203, Tamilnadu, India; 3 Professor and HOD, Department of Anaesthesiology, SRM Medical College Hospital and Research Centre, Potheri, Chennai- 603203, Tamilnadu, India

**Keywords:** TRALI, Hypoxemia, Hemolytic reactions, Microscopic hematuria

## Abstract

**Summary:**

TRALI is a rare but fatal complication of blood transfusion usually under diagnosed and under reported. An interesting case of hemolysis and lung injury developing in a single patient following blood transfusion during perioperative period is reported hereby. Great amount of suspicion about TRALI and supportive care such as mechanical ventilation has saved this patient.

## Introduction

TRALI is transfusion related acute lung injury due to immune mediated reactions between specific leucocyte antibodies and leucocyte antigens, resulting in pulmonary alveolar and capillary membrane damage^1^. It is a life threatening complication of transfusion indistinguishable from the acute respiratory distress syndrome (ARDS) or it's less severe form, acute lung injury (ALI)^2^. It has been reported as the third most common cause of a fatal transfusion reaction^3^. The incidence of TRALI has been reported as 0.16% of all patients transfused though it may be under diagnosed^2^.

## Case report

A 32-year-old male presented with injuries to the right thigh and both the wrists, sustained in road traffic accident. He was diagnosed to have fracture shaft of right femur and Barton fracture both wrists. History and clinical examination revealed no other abnormalities. He was taken up for IM nailing right femur and ORIF both wrists with buttress plating. Patient was assessed as ASA I.

General anaesthesia was planned as both upper limbs and right lower limb have to be operated at one sitting. Patient was premedicated with pethidine 50 mg and glycopyrrolate 0.2 mg iv, preoxygenated with 100% O_2_ for 3 min and induced with thiopentone 250 mg. Patient was intubated with succinylcholine 100mg iv Portex oral cuffed ETT I.D. 8.5mm. Anaesthesia was maintained with N2O/O2 66%:33% and isoflurane 0.6%, ventilation was controlled with vecuronium in intermittent doses. Intraoperative period was uneventful. Blood loss was around 1 litre. Patient was replaced with 3 litres of crystalloids and one unit of ‘A’ positive blood. Urine output was 300ml. At the end of surgery patient was reversed with neostigmine 2.5 mg and glycopyrrolate 0.5 mg iv. Patient was extubated after adequate spontaneous ventilation was established and after satisfying the extubation criteria. There was no residual paralysis. He was observed in postoperative recovery room with routine monitoring.

**Fig 1:**
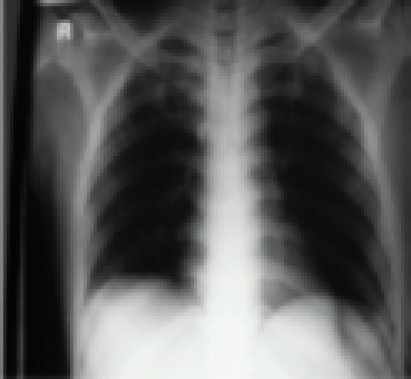
Preoperative CXR

**Fig 2:**
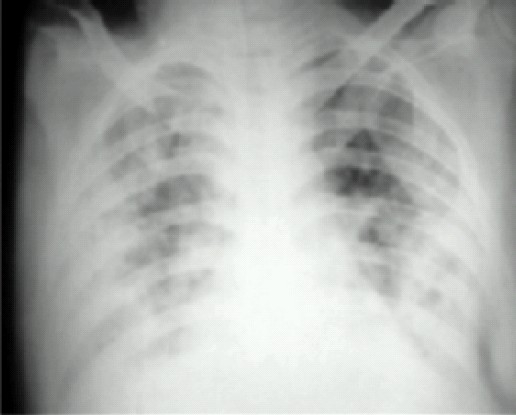
CXR showing bilateral lung infiltrates

Ten min after extubation, patient's SpO_2_ started declining. Since saturation was not maintained above 90% with O_2_ supplementation, patient was reintubated and ventilated with 100% O_2_. Saturation improved to 100% and patient was shifted to surgical intensive care unit for continuous monitoring and mechanical ventilation. All necessary investigations were done. (ECG-sinus tachycardia, ABG- mild hypoxemia, PaO_2_: 76 mmHg). A differential diagnosis of pulmonary/fat embolism, aspiration pneumonitis, pulmonary edema, ARDS were suspected. Ventilatory support continued with FiO2 of 60% and PEEP of 10 cm H_2_O). Chest X-ray showed infiltrates in both lung fields (Fig 2), whereas the preoperative chest X-ray was clear (Fig 1). ETT suction showed blood stained secretions. Right subclavian vein was catheterized and CVP measured which was normal.

**Fig 3:**
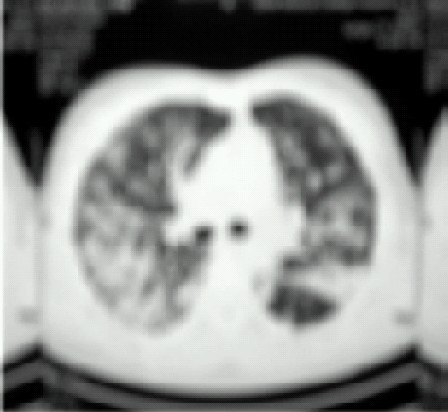
CT picture showing bilateral lung infiltrates

**Fig 4:**
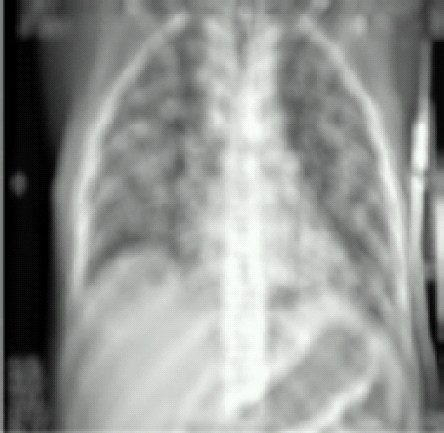
CT picture showing bilateral lung infiltrates

On first postoperative day, patient became icteric. LFT and hemogram were done. Hb decreased to 8 gm% and PCV to 24% (preop Hb 12 gm%, PCV 34%) without any obvious blood loss from wound. Serum bilirubin was increased (Total-4.3, Direct-2.4) and rest were normal. Urine was high colored. Repeat Chest X-ray showed ↑ infiltrates. Urine exam showed microscopic hematuria, peripheral smear showed anemia with neutrophilic leucocytosis with toxic granulations. ABG – metabolic alkalosis, hypoxemia and PaO_2_/FiO_2_ <300. CT scan –ARDS like picture (Fig 3 & 4). Blood culture was sent and TRALI was suspected. Ventilatory support was continued with low tidal volume (6ml/kg), high PEEP (15 cm H_2_O) and high frequency (16/min). Hemodynamic stability was maintained.

Blood sample was sent for regrouping, typing and antibody screen which confirmed A positive.

On second postoperative day there was clinical, radiographic and ABG (PaO_2_/FiO_2_-240) improvement. But Hb ↓ to 6 gm% PCV ↓ to 18%. Urine exam showed ↑ Urobilinogen. One unit of crossmatched whole blood derived from another donor was transfused under supervision and vigilant monitoring.

On third postoperative day, Hb ↑ to 7.2 gm% PCV ↑ to 22%. Serum bilirubin ↑ to 7.8. Microscopic hematuria was present.

On fifth postoperative day, patient showed improvement clinically. Chest X-ray became clear, ABG normal. Blood culture showed no growth.

On sixth postoperative day, patient was weaned and extubated. He was observed in Surgical ICU for 1 week which was uneventful.

## Discussion

TRALI is thought to result from the interaction of specific leucocyte antibodies with leucocytes. Human Leucocyte Antigen (HLA) antibodies, both class I and Class II, and antibodies to Human Neutrophil Antigens (HNA) have all been implicated^1^. Multiparous women have been shown to have a higher rate of HLA sensitization with increasing number of pregnancies^4^ and plasma from multiparous women has been demonstrated to play a part in causing impairment of pulmonary function in a randomized controlled trial^5^. The antibodies are usually donor–derived, though there have been occasional reports of the syndrome occurring after transfusion of donor leucocytes which have interacted with either patient – derived antibodies or antibodies transfused in a second donation, or the presence of HLA antigen/antibody incompatibility in a pooled platelet concentrate. Not all transfusions from donors found to have leucocyte antibodies result in TRALI. Even if there is a match of antigen and antibody specificity, overt lung injury does not always ensue. The transfusion of leucocyte antibodies itself may act as a second “hit”^6^ in a patient whose leucocytes are already primed by other risk factors such as cardiopulmonary bypass or sepsis.

TRALI is clinically indistinguishable from ARDS, which is a type of rapidly progressive and severe respiratory failure that may follow a number of direct and indirect insults to the lung. Post mortem studies show the pathophysiology of ARDS as being one of diffuse damage to alveolar units^7^. Both epithelial and endothelial injury occurs and the alveolar spaces are filled with fluid and proteinaceous debris. Histology shows an intense acute inflammatory cell infiltrate composed of neutrophils and monocytes migrating across the pulmonary vascular bed into the alveolar spaces. The disease is thought to result from intial activation and damage to the pulmonary endothelial/epithelial interface by systemic inflammatory stimuli (both the cellular and circulating mediators) which then stimulate production of further pro-inflammatory mediators and further recruitment of inflammatory cells. ARDS is therefore, the final common presentation following a range of non-pulmonary insults. The question why the lungs are the end organ of choice in TRALI has remained unanswered. A simple explanation may be that where an activating antibody is infused the first microcirculatory encounter is with the narrow diameter capillaries in the lungs. Secondly, the binding of antibodies to mononuclear and polymorphonuclear cells may cause activation partially via the binding of the Fc domain of the antibody to the Fcy receptor and possibly via the activation of complement. Activated granulocytes and monocytes become stickier as adhesion molecules change from their non – active to their active configuration.

TRALI is characterized by symptoms and signs of dyspnoea, cyanosis, hypotension, fever, (none of these is universal) and pulmonary oedema(non cardiogenic). The whole blood count may reveal a leucocytosis, although this may be preceded by leucopenia. The symptoms begin usually within 2 hours after transfusion and well established by 6 hours but may extend up to 24 hours. All blood products including red cells, platelets, plasma, cryoprecipitate and Ivlg^8^ have been reported to cause TRALI.

The problem for clinical diagnosis is that there are no definitive tests and often transfusion has been performed in clinical settings where other causes of acute lung injury are present (e.g. trauma, sepsis). The differential diagnosis is from acute pulmonary edema due to fluid overload/left ventricular failure, or ARDS secondary to other causes. The distinction between TRALI and cardiac failure will be aided by measurement of the left atrial pressure (PAWP) which is typically normal or low in TRALI. A low PaO_2_/FiO_2_ Index (<300 mmHg – Acute Lung Injury, <200 Acute Respiratory Distress Syndrome) is helpful. The development of pulmonary infiltrates on Chest X-rays is not specific. The diagnosis is essentially a clinical one and should be suspected if other reasons to explain the severity of pulmonary edema are not present. Later, investigations for leucocyte antibodies may support the diagnosis.

Hemolytic reactions are one of the most common transfusion reactions which could be either acute due to intravascular hemolysis or delayed due to extravascular hemolysis. The cause of hemolytic reaction is due to ABO/Rh incompatibility mainly or incompatibility of other systems such as kidd, duffy, rarely. The incidence of hemolysis following transfusion of fully crossmatched blood is 1/6000(acute) and 1/1000(delayed). It is mainly due to the misidentification of the patient and blood specimen by the health personnel. Acute hemolytic reactions are very grave in nature, can occur even before 10 ml of blood is transfused and is due to direct attack of transfused donor cells by recipient anti-bodies whereas delayed hemolytic reactions are less serious in nature. The manifestation here is not immediate because the recipient antibody titre is less and they coat the RBCs but do not hemolyse immediately.

The classic features of hemolytic transfusion reactions are chills, fever, chest and flank pain, nausea which are masked by anaesthesia. Under GA, the only signs may be hemoglobinuria, bleeding diatheses and hypotension. Although there are several consequences of intravascular hemolysis, renal and coagulation systems are affected mainly as acute renal failure and DIC respectively

Our patient had all features suggestive of TRALI except hypotension and falling haematocrit (without any blood loss), rising bilirubin and icterus suggestive of delayed hemolytic reactions. However lung injury presented much earlier (within an hour of transfusion) than hemolysis. Patient had no hypotension, no hemoglobinuria or bleeding diatheses at the time of presentation of lung injury. Only on first postoperative day patient had falling haematocrit, rising serum bilirubin and icterus which clearly suggests it is delayed hemolytic reaction and the lung injury was not due to hemolysis. As PaO2/FiO2 was between 200-300, ARDS due to hemolysis or any other cause was ruled out. As the CVP was normal and hemodynamic stability was maintained pulmonary edema was ruled out. Since ECG was normal and no hypotension was present we ruled out pulmonary embolism also. Since blood culture showed no growth sepsis was ruled out. All these raised the suspicion towards TRALI.

## If TRALI is Suspected

- Request samples from the patient
- Samples of donations/donors need to be investigated (initially those from female donors, then male donors whose blood was transfused within 6 hours prior to the onset of TRALI – if these investigations give negative results but TRALI is strongly suspected clinically, investigation should be extended to include donations transfused during the 24 hours prior to the reaction)
- Withdraw and/or recall other components from same donation
- Temporarily suspend the donor(s) pending investigation results

The investigations aim to identify the presence of leucocyte antibodies (HLA class I, HLA class II, HNA in the patient and implicated donor samples. If leucocyte alloantibodies are detected then appropriate test for the presence/absence of the antigen or allele in the patient/donor will be performed. We could not do any of these investigations as the facilities were not available in our set up.

The interpretation of the laboratory results must be in the context of a well – documented clinical case history. If leucocyte antibodies with an obvious allo – specificity are detected in a donor sample and the patient tests positive for the cognate allele or alloantigen then a cross match is not required. Sometimes HLA or other leucocyte antibodies are detected to which no clear specificity can be assigned. In such cases, a crossmatch of donor serum with recipient leucocytes should be performed to confirm incompatibility. A fresh 20ml EDTA blood sample will need to be obtained once the recipient has recovered from the acute phase of TRALI (returned to normal ward, without assisted ventilation) and after all the donor samples have been collected at the testing laboratory. (Note the transfusion within the previous 10 days is a contraindication to performing crossmatch studies because leucocytes can become activated following transfusions).

There is no specific treatment for TRALI. If the transfusion is still continuing, it should be stopped and oxygen and supportive therapy started. If transfusion is unavoidable packed RBCs and blood from new male donors may be considered. As with ARDS/ALI from other causes the precipitating cause should be removed as soon as recognised. Most cases require mechanical ventilation for several days. Appropriate cardiovascular support should be given. Steroids have been advocated but proof of efficacy is lacking.
